# Wind Gust Measurement Techniques—From Traditional Anemometry to New Possibilities

**DOI:** 10.3390/s18041300

**Published:** 2018-04-23

**Authors:** Irene Suomi, Timo Vihma

**Affiliations:** Meteorological Research, Finnish Meteorological Institute, P.O. Box 503, FI-00101 Helsinki, Finland; timo.vihma@fmi.fi

**Keywords:** wind gust, anemometer, Doppler lidar, research aircraft

## Abstract

Information on wind gusts is needed for assessment of wind-induced damage and risks to safety. The measurement of wind gust speed requires a high temporal resolution of the anemometer system, because the gust is defined as a short-duration (seconds) maximum of the fluctuating wind speed. Until the digitalization of wind measurements in the 1990s, the wind gust measurements suffered from limited recording and data processing resources. Therefore, the majority of continuous wind gust records date back at most only by 30 years. Although the response characteristics of anemometer systems are good enough today, the traditional measurement techniques at weather stations based on cup and sonic anemometers are limited to heights and regions where the supporting structures can reach. Therefore, existing measurements are mainly concentrated over densely-populated land areas, whereas from remote locations, such as the marine Arctic, wind gust information is available only from sparse coastal locations. Recent developments of wind gust measurement techniques based on turbulence measurements from research aircraft and from Doppler lidar can potentially provide new information from heights and locations unreachable by traditional measurement techniques. Moreover, fast-developing measurement methods based on Unmanned Aircraft Systems (UASs) may add to better coverage of wind gust measurements in the future. In this paper, we provide an overview of the history and the current status of anemometry from the perspective of wind gusts. Furthermore, a discussion on the potential future directions of wind gust measurement techniques is provided.

## 1. Introduction

Wind gusts are short duration (seconds) wind speed maxima. Storms with extreme wind gust conditions have a major contribution to damage caused by natural hazards. During 1980–2009, storms were the most expensive natural hazards in Europe, with about a 32% share of overall losses (amounting to about 132 billion euros) and 59% of insured losses (about 71 billion euros) [1]. The costs of even one single storm event can exceed billions of euros. For example, the most significant storms in Europe during 1998–2009, the winter storms Lothar and Martin in late December 1999 and Kyrill in January 2007, caused overall losses of 15.5 billion and 7.7 billion euros, respectively [1]. Furthermore, summer storms associated with deep convection can cause severe damage as in Moscow on 29 May 2017, where 16 people were killed and more than 200 injured (Reuters: https://www.reuters.com/article/us-russia-moscow-storm/moscow-storm-death-toll-rises-to-16-idUSKBN18Q1BE, accessed 19 April 2018).

Long-term, continuous, representative wind gust observations form the basis for risk assessments and wind gust forecasting. Estimation of return levels of wind gust maxima in the scale of decades or even centuries are needed for construction planning, but they are also of interest to insurance companies [2]. Wind gust forecasts in the time scales from a day to weeks ahead are useful in preparedness planning and surveillance in different fields of operation such as aviation [3,4], shipping [5] and land transport [6], but also in the energy sector [7,8]. In boreal regions, storms may cause long-lasting and wide-ranging power cuts as trees fall over power lines. For wind energy, information on wind extremes is needed in the planning phase to ensure the strength of the turbine structure [7]. When operating wind turbines, the time scales of interest vary from minutes to multiple days ahead [8]. At shorter time scales, up to minutes ahead, wind information can be used to recognize patterns of oncoming wind speed and/or direction changes, which may lead to extreme load and cause enhanced fatigue or even break turbine components [9,10,11].

To measure wind gusts, a high temporal resolution of the measurements is needed. This means that the whole measurement chain including the anemometer, data acquisition, processing, recording and reporting has to support this. In the dawn of modern anemometry, this was a clear challenge, whereas today, anemometer systems can provide the required high resolution. In the following subsection, the history of anemometry from the perspective of wind gust measurements is briefly reviewed.

### 1.1. History of Wind Gust Measurement Techniques

Many anemometer types used today were invented already in the 18th and 19th Centuries [12]. The first known anemometer was proposed by Leone Battista Alberti in Italy already in 1450. It was a swinging-plate anemometer, which consisted of a disc hanging at the end of a vertical shaft. The disc was placed perpendicular to the wind, and the wind speed measurement was based on the angle between the disc and its original position [13]. In 1667, Robert Hooke developed a similar device, called Hooke’s anemometer. In 1744, Roger Pickering proposed a similar device without knowing these earlier versions of the anemometer. His anemometer consisted in addition of a spring catch that showed the maximum force caused by the wind [12]. This instrument probably provided the first measurement of wind gusts ever. Pressure plate anemometers were still used during the first half of the 20th Century [14,15], but after that, other instruments have taken over.

Another category of pressure anemometers is comprised of pressure tubes. The first one was introduced by Pitot in 1732 to measure flow velocity in water. Following Bernoulli’s theoretical work (1738) on relating the flow velocity to the difference between the total and static pressure in the tube, it was possible to measure velocities using the Pitot tube both in liquid and gas [16]. In meteorology, the pressure tube anemometer was not applied until 1775, when Lind introduced his anemometer. It consisted of a U-shaped tube, whose one limb was bent over towards the wind. By mounting this limb to a vertical spindle, the U-tube formed a direction vane. After Lind, different types of pressure-tube anemometers were introduced, but their difficulty was in the recording. Only the mean wind speed could be obtained as the fluctuating wind speed caused only very small changes in the scale of the manometer. The first self-recording pressure tube was introduced by Dines in 1892. It included pens that recorded both the wind speed and direction. For the first time, it was possible to have also records of wind gusts and lulls, although there were difficulties in obtaining good pen-marking at high wind speeds and in very gusty conditions [16]. Dines pressure tube required a stable surface, and therefore, it was not possible to use it for example at sea. In 1936, Graham introduced a differential aneroid recorder to obtain a more complete record of gusts and lulls than by the float recorder of Dines pressure tube [16]. Today, pressure tube anemometers are mainly used in aircraft and in wind tunnels as reference measurement systems. In aircraft, the most widely-used pressure tube anemometer is the five-hole probe, which can provide high-frequency measurements of all three wind velocity components when the orientation and the speed of the aircraft are known.

Like pressure anemometers, the first rotating anemometers were also introduced in the 18th Century [12]. In the beginning, they resembled wind mills. In 1846, Robinson introduced the first cup anemometer [17], which is similar to those still in wide use in the fields of meteorology and especially in wind energy. The Robinson’s cup anemometer consisted of four cups, but later, a three-cup version became more popular because of its aerodynamic characteristics [16]. The size, shape and design of the cups and the arm lengths and the respective aerodynamic effects on the rotation characteristics have received much theoretical and experimental focus (e.g., [18,19]), and methods to better understand the anemometer performance and dynamic response to wind speed fluctuations are still being developed further (e.g., [20,21]). Over the years, the cup anemometers have achieved an accuracy acceptable for wind energy [22,23], where the accuracy is highly important as the wind power is proportional to wind speed cubed [7].

The first acoustic anemometers were not based on the speed of sound in the air, but were rather composed of an organ that produced different tones with varying wind speed and direction [12]. The interpretation of such measurements was not trivial, and still, in 1941, Sheppard commented that the class of acoustic anemometers is “of no practical importance” [16]. However, already at the end of the 1940s, the first instrument to measure temperature based on the propagation velocity of the sound was developed [24], and in 1955, Schotland presented the theory to measure wind velocity by sonic means [25]. Kaimal and Businger (1963) developed a sonic anemometer that was able to measure both wind and temperature and thereby the eddy heat flux using a vertically-aligned acoustic array [26]. They used two different frequencies for transmission of signals in the two directions. The use of two-way signals canceled the side-wind effect on the wind measurement that was an inevitable disadvantage of one-way signals [25]. This type of approach is still used in modern sonic anemometers [27], where sound pulses are used instead of continuous waves. When the travel times of two-way sound pulses are measured between three different transducer pairs, called sonic paths, the three-dimensional wind vector can be derived. The direct measurement of the three wind velocity components with a system allowing a high frequency of measurements (up to 100 Hz or even more) revolutionized the atmospheric boundary layer research [18], as for the first time, it was possible to directly measure the turbulent fluxes of momentum and buoyancy using eddy covariance technique. Because of these features, sonic anemometers can also be considered as reference instruments for wind gusts.

In the early days of anemometry, the main challenge was to obtain uniform and comparable information on wind force on ships, because suitable recording techniques did not exist. To overcome this problem, Admiral Sir Fancis Beaufort created his well-known scales for wind and weather measurements. The Beaufort scales were first mentioned in a private log in 1806, but the scales were only adopted for wider use much later: the first U.K. Board of Trade publication, the Barometer Manual, mentioned them in 1862 [28]. The Beaufort scales for wind consist of 12 different classes, which are based on qualitative assessment of conditions the wind is causing on the environment at sea. The scales were later converted to units of wind speed [29]. The scales were actively used on ships for over a hundred year period, until the 1980s, providing thus the longest observational time series of winds [30].

Towards the end of the 20th Century, recording practices and signal processing developed. At weather stations from the 1950s onwards, wind gusts were recorded on anemographs from which they were manually read [31]. The first automatic meteorological observing systems were introduced in the 1980s in order to provide observations from remote locations such as Antarctica [32]. At the end of 1980s, the advances in Radio Frequency (RF) communications and microprocessor technology made it possible to establish automatic networks for mesoscale measurement systems, including tens of weather stations measuring wind and other in situ meteorological parameters [33]. Automatization allowed also the calculation of gust speeds using running means [31]. Today, wind gust observations are no longer limited by data processing, recording or reporting techniques because of powerful computers, efficient means of RF communications and inexpensive data storage resources. Moreover, anemometers have reached such a high accuracy that the limited representativeness of point observations is nowadays a larger problem than the anemometer inaccuracy [29,31].

### 1.2. Present Methods and Remaining Challenges

The World Meteorological Organization’s (WMO) recommendation for optimum condition for wind observation is a 10-m reference measurement height over an open area of at least a few kilometers around the site [29]. In practice, these conditions are hardly ever met; therefore, methods have been proposed to correct for a suboptimal site environment, called exposure correction [34,35,36]. These methods are based on turbulence measurements, but if those are not available, information on gusts is used instead, which means that the correction can only be applied to correct the mean wind speed, not the wind gust speed. According to WMO, the representativity of the measurements, for example over a rough surface, can be improved by adjusting the measurement height [29], but it requires a higher supporting structure or a meteorological mast, which can be costly to build.

**Figure 1:**
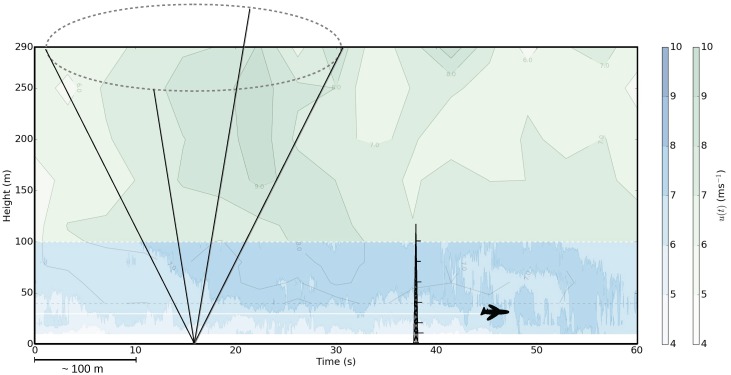
Illustration of the measurement techniques introduced in this study: a Doppler lidar, a meteorological mast and a research aircraft. In the background, an example of a 2D wind field is shown from a Doppler lidar (green) and the sonic anemometers at 10–100 m heights of the mast (blue). The measurements have been collected at the Danish National Test Station for Large Wind Turbines in Høvsøre near the western coast of Denmark. Figure reproduced with permission from [40].

Quite often, the structures of interest for wind gust information are higher than 10 m. Moreover, in many places, the surface is rough, for example over cities and forests, and the measured near-surface wind gusts do not necessarily represent the wind speed maxima at the height of the structures. Strong gust events may encounter a deep layer above the surface: gust fronts associated with deep convection have been observed to cover a depth of about 200 m–2 km above the surface [37]. In situ measurement techniques have limitations in measurement height, but also in areal coverage. The majority of weather stations are located over densely-populated land areas, whereas over remote locations, such as the marine Arctic, observations are made mainly at sparse coastal locations. New techniques to measure wind gusts applying research aircraft [38] and Doppler lidar [39] could fill this gap. In this paper, we discuss these recent developments and the potential new information on wind gusts they will be able provide in the future. The aforementioned aircraft and Doppler lidar measurement techniques are shown in Figure 1. The scanning cone of the Doppler lidar can cover a range of a few hundred meters above the surface, whereas research aircraft can measure over long distances within a short time interval. Both can provide new information on wind gusts that cannot be achieved by meteorological masts alone.

In Section 2, the definition of a gust and how it can be estimated based on statistical theory of maxima is introduced. In Section 3, a brief review of main anemometer types that are presently used for wind gust measurements is included. However, the aim here is not to carry out a thorough review of all wind measurement techniques, but to focus on measurement techniques that can provide valuable information on wind gusts. We present an overview of the remote sensing of wind applying Doppler lidars and Doppler radars in Section 4, although the latter do not represent the temporal and spatial scales relevant for short-duration wind gusts. In Section 5, we will focus on airborne measurement techniques of wind with the main focus on the derivation of wind gusts from turbulence measurements collected on board research aircraft, but we will also briefly discuss the potential of Unmanned Aircraft Systems (UASs) to measure wind gusts. In Section 6, we will review the standard reporting practices of wind gusts, and Section 7 will concentrate on the effect of environmental conditions on wind gust measurements. Finally, in Section 8, a summary is provided with an outlook to the future challenges and opportunities in wind gust monitoring.

## 2. Wind Gust Definition

The wind gust speed, $U_{max}$, is defined as a short-duration maximum of the horizontal wind speed during a longer sampling period (*T*). Mathematically, it is expressed as the maximum of the moving averages with a moving average window length equal to the gust duration ($t_{g}$). Traditionally in meteorological applications, the gusts are measured and the wind forecasts issued using a gust duration $t_{g}=$ 3 s and a sample length T= 10 min [29]. However, sometimes, other durations are used, as well; for example, until the early 2000s wind gusts at the Automated Surface Observing System (ASOS) network of the U.S. National Weather Service (NWS) were based on hourly (T= 1 h) records with $t_{g}=$ 5 s [41,42,43]. Originally, the definition of a wind gust had been based on the response characteristics of old anemometers, whose response times were about 2–5 s [44].

The ratio of the wind gust speed $U_{max}$ to the mean horizontal wind speed *U* is called the gust factor:

(1) $$G=\frac{U_{max}}{U}.$$

Thus, *G* is proportional to the inverse of the mean wind speed. In practice, the measured *G* values show a large variability at low mean wind speeds, but at strong wind speeds (neutral conditions), *G* typically reaches a constant value characteristic of the measurement site. The mean *G* depends on various environmental factors, such as the measurement height, surface roughness, atmospheric static stability (more in Section 7) and on the temporal scales used in the gust definition, the gust duration $t_{g}$ and the sample period *T* [38,45,46].

Another measure of gustiness is the peak factor:

(2) $$g_{x}=\frac{U_{max}-U}{\sigma_{u}},$$

which is the deviation of the wind gust speed from the mean normalized by the standard deviation of the horizontal wind speed ($\sigma_{u}$). Because of the normalization, the peak factor can be considered to be invariant of environmental conditions contrary to the gust factor (Equation (1)), but instead, it is, above all, a function of the gust time scales, $t_{g}$ and *T*. Moreover, the peak factor can be derived from theoretical considerations, as shown below in this section. The peak factor is a useful tool in scaling wind speed maxima from a low-response system to provide wind gust measurements comparable with weather station measurements [39]. Peak factors can also be used for comparison of spatial and temporal scales [38].

The peak factor can be estimated from theoretical considerations. It is based on a statistical approach to estimate the maximum value in the time series when the mean statistical properties (*U*, $\sigma_{u}$, power spectrum) of the wind speed are known. This statistical theory of maxima was first proposed by Rice (1944, 1945) and is therefore called the Rice theory [47,48]. Since then, the theory has been applied in various studies with small modifications. Davenport (1964) applied the Rice theory to wind speed measurements [49]. Greenway (1979) tested the method against wind tunnel measurements and evaluated the effect of filtering on the gust factor to estimate loads on structures [44]. Beljaars (1987) used the method to derive a parameterization for NWP models [50]. His approach is still used in the operational Integrated Forecasting System (IFS) of the European Centre for Medium-Range Weather Forecasts (ECMWF) with a small modification to account for convective gusts [51]. Kristensen et al. (1991) compared the method to the Gumbel theory of extremes and suggested that these theories are equivalent [52], although this is not exactly true. Wichers Schreur and Geertsema (2008) took one step further and combined the Rice theory to include Turbulence Kinetic Energy (TKE) from a weather model when it is available [53]. In [46], the theory was validated against observations from a 100 m-high meteorological mast. Suomi et al. (2016, 2017) applied the method to derive observations of wind speed maxima from instrumentation on board a research aircraft [38] and from a Doppler lidar [39]. Both instrumentations have the potential to provide wind gust measurements from heights and regions yet unreachable by the traditional in situ anemometers.

According to the Rice theory, the probability that the instantaneous horizontal wind speed u(t)=u performs one up-crossing (the time derivative $\dot{u}>0$) of the level $U_{x}$ is:

(3) $$\int_{0}^{\infty }d\dot{u}\int_{U_{x}-\dot{u}\Delta t}^{U_{x}}P\left(u,\dot{u}\right)du\approx \Delta t\int_{0}^{\infty }\dot{u}P\left(U_{x},\dot{u}\right)d\dot{u},$$

where $P(u,\dot{u})$ is the joint probability of the wind speed and its time derivative [52]. The covariance of *u* and $\dot{u}$ is:

(4) $$\langle \left(u-U\right)\dot{u}\rangle =\frac{1}{2}\frac{d}{dt}\langle u^{2}\rangle ,$$

where the angle brackets denote the mean. If the variance of *u* is stationary (does not depend on time), i.e., $\frac{d}{dt}\langle u^{2}\rangle =0$, *u* and $\dot{u}$ will be uncorrelated and hence statistically independent:

(5) $$P\left(u,\dot{u}\right)=P\left(u\right)P\left(\dot{u}\right).$$

Following [52], both probabilities, P(u) and $P(\dot{u})$, are assumed Gaussian:

(6) $$\begin{array}{cc} P(u) & =\frac{1}{\sqrt{2\pi }\sigma_{u}}e^{-\frac{{\left(u-U\right)}^{2}}{2\sigma_{u}^{2}}}\\ P(\dot{u}) & =\frac{1}{\sqrt{2\pi }\sigma_{\dot{u}}}e^{-\frac{{\dot{u}}^{2}}{2\sigma_{\dot{u}}^{2}}}, \end{array}$$

although real atmospheric turbulence is typically non-Gaussian, because higher order statistical moments contribute to the probability density function (e.g., [54]). To estimate those, quasi-Gaussian turbulence models have been developed (e.g., the Quasi-Normal Scale Elimination (QNSE) method by [55,56]), which are, however, beyond the scope of this work. For strong gusts (for which U>>0), the two probabilities in Equation (6) are fairly good approximations.

Following from Equations (3), (5) and (6), the average rate η of up-crossing of the level $U_{x}$ is:

(7) $$\eta \left(U_{x}\right)=P\left(U_{x}\right)\int_{0}^{\infty }\dot{u}P\left(\dot{u}\right)d\dot{u}=\frac{\sigma_{\dot{u}}}{2\pi \sigma_{u}}e^{-\frac{{\left(U_{x}-U\right)}^{2}}{2\sigma_{u}^{2}}}.$$

The expectation value for the number of upcrossings of the level $U_{x}$ within a time series of length *T* is $N\left(U_{x}\right)=\eta \left(U_{x}\right)T$. The probability for upcrossing the level $U_{x}$ *k*-times within the time interval *T* can be estimated by using the Poisson distribution:

(8) $$P\left(k,N\right)=\frac{N{\left(U_{x}\right)}^{k}}{k!}e^{-N(U_{x})}.$$

As we are interested in estimating the maxima of the wind speed time series, we will set k=0, which gives the probability of *u* not exceeding $U_{x}$ in period *T*:

(9) $$P\left(0,N\right)=\exp\left(-N(U_{x})\right).$$

Now, combining Equations (7) and (9) yields:

(10) $$P\left(u<U_{x}\right)=\exp\left(-\frac{T}{2\pi \tau }e^{-\frac{{\left(U_{x}-U\right)}^{2}}{2\sigma_{u}^{2}}}\right),$$

where $\tau =\frac{\sigma_{u}}{\sigma_{\dot{u}}}$ is the turbulent time scale. From Equation (10), we can estimate the peak factor as $g_{x}\approx \frac{U_{x}-U}{\sigma_{u}}$ yielding:

(11) $$g_{x}\approx {\left[2\ln\left\{\frac{T}{2\pi \tau }\frac{1}{\ln\left(\frac{1}{P}\right)}\right\}\right]}^{0.5},$$

where *P* is the probability of a peak factor not exceeding the value $g_{x}$ in an ensemble of samples. That is, $P\left(u<U_{x}\right)=P\left(\left(u-U\right)<\left(U_{x}-U\right)\right)=P\left(\frac{u-U}{\sigma_{u}}<\frac{U_{x}-U}{\sigma_{u}}\right)$. Thus, the median peak factor in an ensemble of samples corresponds to a probability P=0.5.

The turbulent time scale τ in Equation (11) can be estimated using information on the (one-sided) turbulence power spectrum S(f), where *f* is the frequency (e.g., [57]).

(12) $$\tau =\frac{1}{2\pi }{\left(\frac{\int_{0}^{\infty }S\left(f\right)df}{\int_{0}^{\infty }f^{2}S\left(f\right)df}\right)}^{1/2}.$$

The advantage of Rice theory is that it provides the theoretical basis to estimate the relationship between the gust duration and the wind gust speed. Equation (12) provides the turbulent time scale of the continuous wind speed signal including all scales of turbulent motion beyond the sample length *T*.

When a moving average is applied to the time series, the statistical properties of the time series change. This can be described by introducing a filter ${\left|H\left(f\right)\right|}^{2}$ to the spectrum as:

(13) $$S_{f}\left(f\right)={\left|H\left(f\right)\right|}^{2}S\left(f\right),$$

where $S_{f}\left(f\right)$ is the filtered spectrum. The filter function for a traditional cup anemometer instrumentation is:

(14) $${\left|H\left(f\right)\right|}^{2}={\left(\frac{sin(\pi ft_{g})}{\pi ft_{g}}\right)}^{2}{\left(\frac{sin(\pi f\Delta t_{o})}{\pi f\Delta t_{o}}\right)}^{2}\frac{1}{1+{\left(2\pi f\frac{l}{U}\right)}^{2}}$$

where the first component on the right-hand side is the moving average filter with an averaging window length of $t_{g}$, which is the gust duration. The other two components are typically used when the aim is to describe the effect of the measuring chain on the resulting wind speed time series [44,50]. The second component on the right-hand side of Equation (14) expresses the effect of sampling frequency $f_{o}$, where the interval between individual measurements $\Delta t_{o}=1/f_{o}$. The last component on the right-hand side is the filtering caused by the anemometer inertia in the case of a cup or a propeller anemometer with a response length *l*. Anemometer response depends on the mean wind speed: at low wind speeds, a cup/propeller anemometer responds slower than at high wind speeds. The response of a sonic anemometer to wind speed variations depends mainly on the dimensions of the instrument, because for a typical instrument setup, the sampling frequency is 10–20 Hz, i.e., $\Delta t_{o}<<t_{g}$. The effect of anemometer dimensions on the measured turbulence can be estimated in the same way as the third component on the right-hand side of Equation (14). However, this component is less important for sonic anemometers than for cup anemometers, because the dimensions of a sonic anemometer (i.e., the length of sonic paths) are typically small, of the order of about 10–20 cm, whereas for a typical cup anemometer, the response length is l≈ 2 m. Moreover, for most applications, wind gust measurements are meaningful only at high wind speeds, when l/U is typically small.

After filtering (replacing S(f) in Equation (12) by $S_{f}\left(f\right)$ from Equation (13)), the resulting peak factor from Equation (11) provides an estimate of the peak of the filtered (moving averaged) wind speed relative to the standard deviation of this filtered signal. To estimate the gust factor using Equation (11), we are interested in the peak factor relative to the true turbulence, not that of the filtered signal. Therefore, we need to multiply the peak factor from Equation (11) by an additional coefficient, $r_{\sigma }$, which is the ratio of the filtered and the true turbulence:

(15) $$r_{\sigma }={\left(\frac{\int_{0}^{\infty }{\left|H\left(f\right)\right|}^{2}S\left(f\right)df}{\int_{0}^{\infty }S\left(f\right)df}\right)}^{1/2}.$$

The theoretical peak factor method presented above can be applied to convert between different gust time scales if the gust measuring practices differ [46], but also to convert between temporal and spatial scales [38]. How this theory can be applied to obtain wind gust measurements from Doppler lidar and research aircraft is shown in Section 4 and Section 5. Nevertheless, an overview of surface-based in-situ wind gust measurement techniques is included in Section 3, as all existing long-term wind gust records are based on such techniques.

## 3. Surface-Based In-Situ Measurement Techniques

Wind gusts are observed routinely at WMO coordinated weather stations all over the world using traditional in situ measurement techniques. In this section, we provide a brief review of the main anemometer types focusing on their aspects that are important for measuring wind gusts. Anemometers are divided into groups of rotating anemometers, sonic anemometers, pressure anemometers and anemometers based on the cooling rate caused by the wind.

### 3.1. Rotating Anemometers

**Figure 2:**
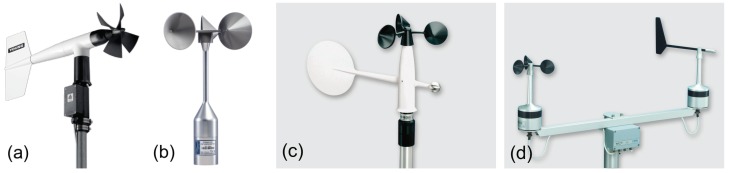
Examples of rotating anemometers, from the left: (**a**) propeller anemometer Wind Monitor Model 05103 by R. M. Young Company; (**b**) cup anemometer P2546-OPR by WindSensor; (**c**) combined cup-vane anemometer Wind Sensor WM30 by Vaisala; and (**d**) cup anemometer and wind vane WA25 Wind Set by Vaisala.

Rotating anemometers comprise cup and propeller anemometers (Figure 2). The latter is typically a combination of a propeller and a wind vane, providing both the wind direction and speed. Furthermore, cup anemometers have been introduced as combined systems with a wind vane [19], although more often, they are deployed as separate instruments.

The cup anemometer is an old but thoroughly tested technology that, even more than 150 years after its design by Robinson, is being improved via new research [58]. Cup anemometers have many advantages compared to other instruments. They are robust; the measurement accuracy is high; they are easy to apply, relatively inexpensive and can be run over long periods without maintenance and/or recalibration. Cup anemometer calibration is a well-known procedure [22,23]. The measurement principle is simple: the angular velocity (ω) of the anemometer is a linear function of wind speed over most of the wind speed range (except close to the starting speed, which is nowadays typically less than 1 ms${}^{-1}$, i.e., practically unimportant for wind gusts) [19]. The anemometer factor:

(16) $$f_{a}=\frac{u}{r\omega },$$

where *r* is the cup anemometer rotor radius (distance from the axis of rotation to the center of a cup) and ω the rotor rotation rate. $f_{a}$ depends on the design of the anemometer and varies typically from 2.5–4.5 between different anemometer types (e.g., [19,21]).

Although the cup anemometer calibration is simple and linear, its dynamic response to wind fluctuations is highly non-linear (e.g., [18,19,20,21,59]), i.e., the anemometer responds faster to a wind speed increase than to a decrease. This effect is mainly caused by the lateral wind velocity component (horizontal wind component perpendicular to the mean wind direction) [19], and it affects mostly the mean wind speed when averages are taken over long periods: the asymmetry of the response causes a positive bias, called overspeeding, in the resulting mean wind speed. However, we depict that overspeeding is less important for wind gusts than for the mean wind speed, as the wind gusts represent an average over a few seconds only. Instead, the more important parameter for wind gusts is probably the response length *l* of the cup anemometer (introduced in Equation (14)). It is the length of the air volume that has to blow through the anemometer for this to reach $1-e^{-1}$≈ 63% of its final reaction to the change in wind speed. Like anemometer factor $f_{a}$, it depends on the anemometer design, but also on the density of the rotor material in relation to air density ($\rho /\rho_{a}$): rotors made of heavy material react slower than light-material anemometers. Moreover, as *l* is inversely proportional to air density ($\rho_{a}$), the instrument may react 10–20% slower at higher altitudes compared to that at the sea level [19]. Cup anemometers used at weather stations (Figure 2) have a response length *l* of about 2 m, which means that at wind speeds of 10 ms${}^{-1}$, the cup anemometer measurements are sensitive at time scales down to 0.2 s, i.e., the measurements are representative of wind gusts when the gust is calculated as an average over 3 s. At very low wind speeds, the anemometer response may affect short averages of wind speed, but on the other hand, the main aim of measuring wind gusts is not for weak winds.

The dynamics of a propeller anemometer resemble that of a cup anemometer with a linear calibration and a non-linear response to wind speed fluctuations. However, the overspeeding is less serious for propeller anemometers than for cup anemometers [18].

### 3.2. Sonic Anemometers

**Figure 3:**
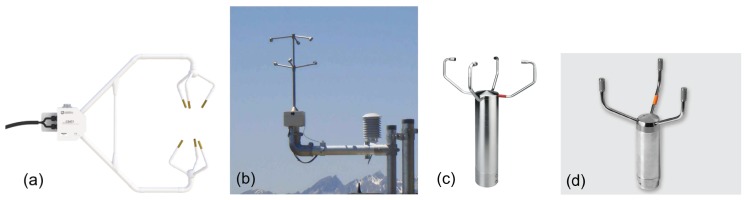
Examples of (ultra)sonic anemometers, from the left: (**a**) CSAT3 3D sonic anemometer by Campbell Scientific, Inc.; (**b**) uSonic-3 Scientific (former: USA-1) 3D sonic anemometer by METEK GmbH; (**c**) Ultrasonic Anemometer 2D by Thies Clima; and (**d**) WINDCAP 2D Ultrasonic Wind Sensor WMT700 by Vaisala.

Sonic anemometers have become standard instruments in micrometeorological research and monitoring today (e.g., [60]). They use ultrasonic frequencies and pulse repetition rates of the order 50–100 Hz, which are then block averaged to provide output frequencies of 1–20 Hz. Moreover, different types of sonic anemometers have been introduced, with one (provides only one wind component), two (for horizontal wind) or three measurement axes (provides the full 3D wind vector), which can be either orthogonal or non-orthogonal, the latter being more common in 3D sonic anemometers nowadays (Figure 3). Wind velocity component(s) are derived from the composition of travel times of sound pulses along the sonic axes. The wind velocity component along one sonic path is:

(17) $$v_{s}=\frac{d}{2}\left(\frac{1}{t_{1}}-\frac{1}{t_{2}}\right),$$

where *d* is the length of the sonic path and $t_{1}$ and $t_{2}$ are the travel times of the sound pulse in the two directions along the sonic path. The speed of sound can be approximated as:

(18) $$c_{0}\approx \frac{d}{2}\left(\frac{1}{t_{1}}+\frac{1}{t_{2}}\right).$$

However, when there is a wind component perpendicular to the sonic path, a correction for the cross-wind component ($v_{cw}$) must be introduced. The true speed of sound is then:

(19) $$c=\sqrt{c_{0}^{2}-v_{cw}^{2}}.$$

Some of the sonic anemometers perform the cross-wind correction internally (e.g., CSAT3 by Gampbell Scientific, Inc., Logan, UT, USA), but in some configurations, it must be applied afterwards [61]. The sonic temperature can then be derived from the speed of sound as:

(20) $$T_{S}=\frac{c^{2}}{\gamma_{d}R_{d}},$$

where $\gamma_{d}=$ 1.4 and $R_{d}$ is the gas constant for dry air ($R_{d}=$ 287.04 JK${}^{-1}$kg${}^{-1}$), giving $\gamma_{d}R_{d}\approx$ 403 m${}^{2}$s${}^{-2}$K${}^{-1}$ [61,62]. Sonic temperature is given by $T_{S}=T\left(1+0.51q\right)$ [62], where *T* is the true air temperature and *q* the specific humidity. It is noteworthy that $T_{s}$ is very close to the virtual air temperature $T_{v}=T\left(1+0.61q\right)$ [63]. If a sonic anemometer is combined for example with a fast-response hygrometer or an infrared gas analyzer, the turbulent fluxes of heat and moisture, greenhouse gases and/or other contaminants can be measured (e.g., [60,61,62,64]).

Sonic anemometers are in many ways superior to other instrumentations used in turbulence measurements. The wind velocities are measured directly, so there is no anemometer inertia involved in the measurement. The high measurement frequencies allow good coverage of the turbulent fluctuations of all scales. The response characteristics are only limited by the length of the sonic paths, which are typically about 10–20 cm only, an order of magnitude smaller than the response length of cup anemometers (Section 3.1 and [18]). Therefore, sonic anemometers are suitable for reference measurements for wind gusts, as well. However, there are several sources of error for sonic anemometers, as well. Transducers may affect the measurement accuracy by causing a wake along the sonic path, and therefore, the absolute values of the measured wind may be biased low [65,66]. Flow distortion can also be caused by the support struts of the transducer array [66]. A proper design of the anemometer, by optimizing the ratio of the diameter of the transducer head to the sonic path length and by alignment of the sonic paths with a large angle with the horizontal, can reduce the wake effects on the measured wind. Different designs of support struts have also been introduced, as seen in Figure 3. The flow distortion effects are usually corrected by built-in calibration tables [66]. Sonic anemometers are also sensitive to temperature changes, which can affect the measurement of the travel times along the sonic paths [66]. In addition to these known error sources, sonic anemometers may have challenges in rain and snow; these conditions can affect the propagation of sound pulses.

Sometimes, instruments internal errors result in unrealistically high (or low) values in the measured time series, called spikes. Even only one such value in the time series may lead to overestimation of the wind gust speed. Methods, called spike detection and removal, have been proposed to correct for these outliers [39,67,68,69]. They are based on two-point statistics of the time series, using information about the previous values in the time series to provide a forecast for the next value. If the magnitude of the next observed value is several standard deviations away from the forecasted one, the observed value is considered as a spike and removed from the time series for example by linear interpolation using neighboring non-spike values. The method performs well when there are only a few outliers, but deteriorates when unrealistic values start to dominate the time series [39]. Consecutive anomalous values may sometimes be caused by real atmospheric phenomena. To account for that, a criterion is introduced that if there are at least four consecutive detected values in row, the values are not considered as spikes, but as a physical feature of the flow [68,69].

### 3.3. Pressure Anemometers

Pressure anemometers can be divided into groups of pressure plate anemometers and pressure tube anemometers. As pointed out in Section 1, pressure plate anemometers have not been used in meteorological applications since the first half of the 20th Century, mainly because of their complex dynamic response to wind speed fluctuations. Instead, pressure tube anemometers, called Pitot tubes, are widely used in meteorological research, especially on board aircraft (five-hole probes) and in wind tunnels. The basic Pitot tube is an L-shaped tube consisting of two pressure ports and the sensing tip [70]. The differential pressure transducer provides as output the difference between the total and static pressures, which represents the dynamic pressure caused by the wind. In other words, the pressure difference is proportional to the wind speed squared. The Pitot tube equation for a standard instrument type and for incompressible flow (small Mach number) is:

(21) $$v_{p}=44.72136\sqrt{\frac{h_{\mathrm{kPa}}}{\rho_{a}}},$$

where $v_{p}$ is the measured wind speed, $h_{\mathrm{kPa}}$ is the pressure difference from the transducer and $\rho_{a}$ is the air density, which is estimated based on barotropic pressure and the absolute air temperature measurements from parallel instruments [70]. In addition, $\rho_{a}$ can also be corrected for the effect of water vapor content, if relative humidity is measured. However, the magnitude of this correction is typically of the same order as the accuracy of the instrument [70].

The Pitot tube calibration is complex because of the non-linear relationship between the (dynamic) velocity pressure and the air flow rate. The calibration has to be done in a wind tunnel and is typically based on a look-up table that provides the ratio of the true flow rate and the measured flow rate. This ratio varies by the tube design and for example by inflow angles. In case of a five-hole probe, typically used on board aircraft to measure the 3D wind vector, the calibration is extremely complicated, and various calibration algorithms have been introduced (e.g., [71]). However, the advantages of the Pitot tube are the high frequency of the measurements, very good accuracy and an almost linear calibration for strong wind speeds, which makes it suitable for aircraft measurements, where the true air speed is high. However, in laboratory conditions in wind tunnels, where even very small pressure differences can be measured accurately, it can provide accurate turbulence measurements [19]. Pitot tubes have not been used much to investigate wind gusts, but they can provide wind gust measurements when used on board aircraft (Section 5, [38]).

### 3.4. Anemometers Based on the Cooling Rate

Hot wires have been used for wind measurement since the beginning of the 20th Century [16,72]. The measurement principle is based on the cooling effect of the wind on a heated wire. These instruments are the most sensitive to wind fluctuations and can provide measurements with a high precision, especially at low wind conditions. However, their disadvantage is that they are very fragile and therefore not suitable for strong winds and long-term measurements for example at weather stations; therefore, they are not preferred instruments for measuring wind gusts. Typically, hot wires are used indoors and in wind tunnels, where a high precision of the wind measurement is needed. Another disadvantage of hot-wire anemometers is their sensitivity to temperature changes, and therefore, they require frequent calibration [73].

## 4. Remote Sensing Techniques

Remote sensing of wind is based on the Doppler technique, where the wind velocity component in the direction of the measurement signal is derived from the Doppler shift between the transmitted and the backscattered signal. The same technique is applied in Doppler lidar (light detection and ranging), Doppler sodar (sonic detection and ranging) and Doppler radar (radio detection and ranging). The known travel (half) time provides the distance range for the measurement and the obtained velocities represent the average velocity of the particles/air parcels from which the signal is backscattered. The backscattering depends on the wavelength used. For radar, backscattering takes place mainly from hydrometeors, but also from insects and birds [74]. Sodar echos on the other hand return from inhomogeneities in air density caused by turbulence and from vertical gradients in temperature and wind (e.g., [75]). lidar backscattering takes place from aerosols and other small particles in the air [76]. In order to measure wind gusts, only lida can provide high enough temporal and spatial resolution. Therefore, in this section, we will focus on Doppler lidar measurements. However, Doppler radar can potentially provide valuable information on convective gust fronts, and therefore, they are briefly discussed at the end of the section.

### 4.1. Doppler Lidar

Doppler lidar technology to measure wind is fairly new. The first commercial small-sized (<100 kg) Doppler lidars appeared on the market only about 15 years ago [76]. Today, they are widely applied for example in wind energy research [77,78], and networks of continuous Doppler lidar measurements are being established (e.g., EU/ESSEM COST Action ES1303: “Towards operational ground based profiling with ceilometers, Doppler lidars and microwave radiometers for improving weather forecasts” (TOPROF)). However, the field is still undergoing fast developments, and new methods to apply the technique are introduced continuously.

**Figure 4:**
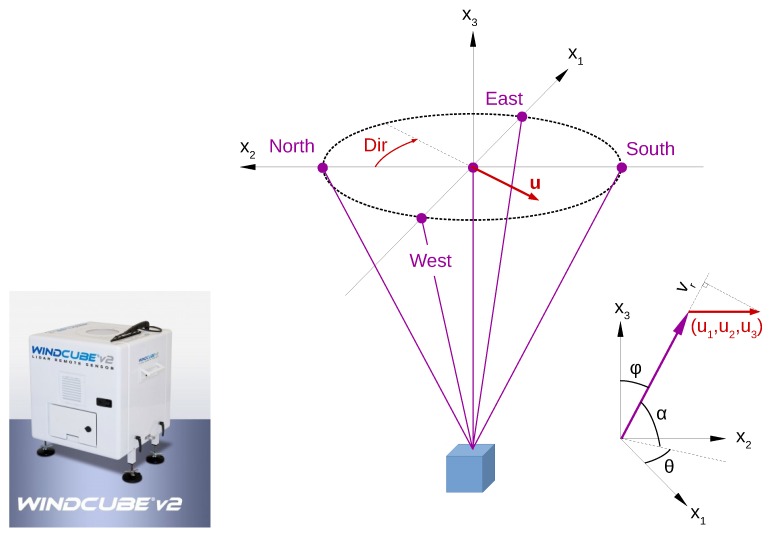
WindCube V2 Doppler lidar by Leosphere measures along five lines of sight, one vertical and four inclined with a fixed zenith angle φ = 28${}^{\circ }$ (elevation angle α = 62${}^{\circ }$) and with an azimuth angle of θ = 90${}^{\circ }$ between the inclined beams. Using radial velocities $v_{r}$ along at least three different beams, it is possible to derive the three-dimensional wind vector $\left(u_{1},u_{2},u_{3}\right)$ [79,84].

The basic principle of a Doppler lidar is that the instrument transmits a laser beam (either a continuous wave or a pulse) to the atmosphere and then receives a small part of the signal backscattered from aerosols and other particles flowing in the air along with the wind. From the Doppler shift between the transmitted and received signal, it is possible to derive the radial wind speed component ($v_{r}$) along the laser beam (Figure 4). The radial wind speed can be expressed in terms of the three orthogonal wind vector components ($u_{1}$, $u_{2}$, $u_{3}$) and the zenith (φ) and azimuth (θ) angles of the lidar line of sight

(22) $$v_{r}\left(\theta ,\varphi \right)=u_{1}\cos\theta \sin\varphi +u_{2}\sin\theta \sin\varphi +u_{3}\cos\varphi .$$

When combining information from at least three different lines of sight ($v_{r_{1}}$, $v_{r_{2}}$, $v_{r_{3}}$), it is possible to obtain the three-dimensional wind vector ($u_{1}$, $u_{2}$, $u_{3}$). The majority of Doppler lidars today are operated as single-lidar systems, but also set-ups consisting of two or three lidars have been introduced [78]. A single Doppler lidar set-up can be used with various scanning strategies (e.g., [78,79]):

- Doppler Beam Swinging (DBS) scan consists of 3–5 beams, of which one can be vertical (three- and five-beam systems) and others inclined with a fixed elevation angle (α) of about 60–70${}^{\circ }$, and an azimuth angle (θ) of 90${}^{\circ }$ (or 270${}^{\circ }$) between the beams. An illustration of a DBS scanning pattern with five beams is shown in Figure 4.
- Velocity-Azimuth Display (VAD) scan has many inclined beams with a fixed azimuth angle between neighboring beams and a fixed elevation angle that can be chosen by application. With very low level (small elevation angle) scans, it is possible to observe the horizontal variability of the wind, and with a higher elevation angle, more accuracy can be achieved for the measurement of mean wind speed profiles [80].
- The Range Height Indicator (RHI) scanning technique applies a fixed azimuth angle, and the elevation angle changes between the beams. This type of scan, when applied to the direction of the incoming wind, can be used to detect anomalies in a turbulent wind field [81,82,83].
- Plan Position Indicator (PPI) scan varies the azimuth angle with a fixed elevation angle and therefore takes measurements on a conical surface.
- Nacelle-based Doppler lidars deployed on top of a wind turbine hub can provide valuable information on the incoming wind [11,76]. The scan patterns can be complicated with respect to providing the best possible coverage and availability in the scale of the rotor disc swept by the blades [79].

**Figure 5:**
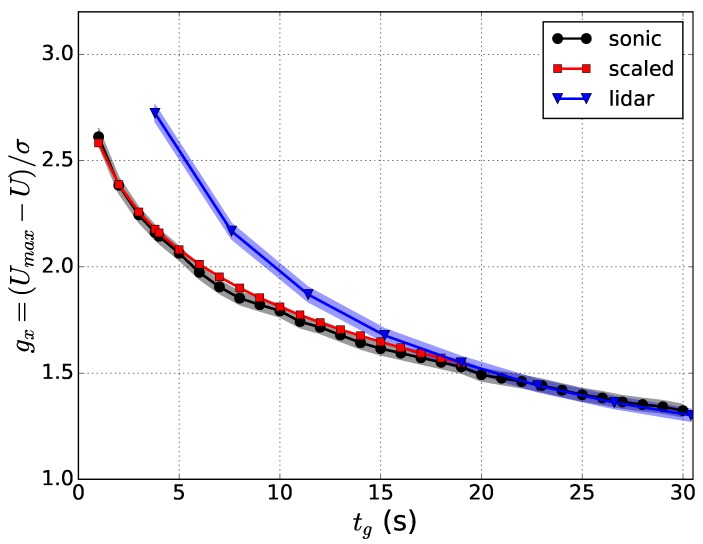
Median peak factor as a function of gust duration as observed by sonic anemometers (black), lidar (blue) and the theoretical peak factor (red) derived from the parallel measurements from lidar and sonic anemometers. All peak factors are normalized by the standard deviation of the wind speed from the sonic anemometers. The standard error of the mean is given by the shadowed region underlying the points in each median curve. Redrawn with permission from [39].

The Doppler lidar technique can provide various possibilities to investigate wind variability both in time and in space. The instrumentation is easy to apply, relatively inexpensive and measurements can reach high altitudes, even above the tallest meteorological masts. Therefore, for example in wind energy, Doppler lidar can be a good alternative to meteorological mast measurements, especially in environments where traditional meteorological masts cannot be built or their deployment is challenging or costly, for example offshore. Doppler lidar measures the mean wind speed with a high and known accuracy (e.g., [69,85,86]). For turbulence measurements, Doppler lidar scanning frequency and scanning strategies pose limitations, and methods to estimate turbulence parameters have been developed [77]. The same limitations apply also for wind gust measurements. Suomi et al. (2017) [39] used a short-range (measurements up to 300 m height) pulsed Doppler lidar to obtain wind gust profiles. The DBS scanning technique consisted of five beams, one vertical and four inclined with θ= 90${}^{\circ }$ azimuth angles and an elevation angle of α= 62${}^{\circ }$. The resolution of wind speed observations was 3.8 s, which is the time it takes to measure all five beams. The observed wind speed maxima from the Doppler lidar were clearly higher than those from sonic anemometers at a nearby meteorological mast (the difference between the blue and black curves in Figure 5). Suomi et al. (2017) [39] developed a scaling method to correct for this positive bias, which is shown as a red curve in Figure 5. This novel method not only scales the lidar gusts, but also provides estimates for wind gusts with variable gust durations including the shorter durations (of the order of a second), which are beyond the limits of the lidar measurement frequency. The input parameters for the scaling method include the wind gust speed, mean wind speed and the standard deviation of the horizontal wind speed from the lidar. The wind gust speed is calculated as the maximum of the moving-averaged horizontal wind speed. For the WindCube V2 Doppler lidar used by [39], an average over five samples (corresponding to gust duration $t_{g}=$ 19 s) was found to be adequate, but this depends on the lidar type and the scanning technique and must be tuned separately for each lidar setup. Using Doppler lidar data only for the scaling method will provide reasonable gust factor estimates, with a small positive bias (about 0.03) and RMSE of about 0.06, but it is possible to further reduce this bias by better estimates of the velocity variance. The improved performance of the scaling method was most noticeable in turbulent daytime conditions, but it also improved the estimation of gustiness in precipitating conditions, which are a challenge for Doppler lidars.

In the case of Doppler lidars, data quality is traditionally assessed based on the Carrier-to-Noise-Ratio (CNR). However, Suomi et al. [39] found that better results were obtained with a spike detection algorithm typically used for sonic anemometer data. The algorithm effectively removes outliers (including unrealistically high values) that may arise for example from incorrect instrument’s internal calculation of CNR [87]. The outliers (the spikes) were replaced by linear interpolation using neighboring non-spike values.

**Figure 6:**
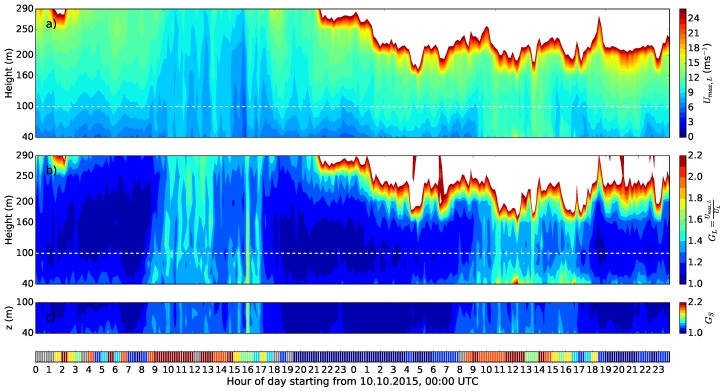
Time–height cross-sections of (**a**) the wind-gust speed ($U_{max,L}$) and (**b**) gust factor ($G_{L}$) from the lidar measurements after applying the despiking procedure. For comparison, the gust factors ($G_{S}$) from the meteorological mast are shown in (**c**). The bottom panel shows the stability conditions based on the inverse of the Obukhov length during each 10-min sample: dark red: very unstable (−0.01 $>L^{-1}\geq$ −0.02), orange: unstable (−0.005 $>L^{-1}\geq$ −0.01), yellow: near neutral unstable (−0.002 $>L^{-1}\geq$ −0.005), green: neutral ($|L^{-1}|\leq$ 0.002), light blue: near neutral stable (0.005 $\geq L^{-1}>$ 0.002), blue: stable (0.02 $\geq L^{-1}>$ 0.005) and dark blue: very stable (0.1 $\geq L^{-1}>$ 0.02). The criteria for stability groups are based on [88]. Redrawn with permission from [39].

An example of the wind gust speed and the gust factor measured by a Doppler lidar during a two-day period is shown in Figure 6. During the first day, good data were obtained through the entire measurement range, but on the second day, when there were rainy conditions, the data availability was limited. The mean gust factor profiles of the first day in Figure 6 are shown in Figure 10 separately for unstable, near neutral and stable conditions. Gust factors from the lidar match those from the meteorological mast in neutral and stable conditions, but in unstable conditions, they are overestimated at the 60 m level and above. As the lidar combines data from four–five lines of sight to construct the 3D wind vector, the heterogeneity of the wind field at the scale of the cone defined by the lidar measuring beams (scales of about 100–300 m in this case) propagates to the wind vector estimate. In turbulent daytime conditions, where the wind is strongly variable, the measured wind gusts are affected by these wind fluctuations, which leads to the overestimation of the gust factor when compared to pointwise measurement from the meteorological mast.

### 4.2. Doppler Radar

Doppler radar wind measurements do not actually represent the temporal and spatial scales of wind gusts as defined here, because the Doppler radar resolution is of the order of 200–300 m. However, the advantage of Doppler radars is the long measurement range (up to about 150 km) and the availability of measurements from rainy conditions, which are a challenge for Doppler lidars, as seen on the second day in Figure 6. Hence, a Doppler radar can be an essential tool in nowcasting approaching gust fronts related to deep convection. Gust fronts can indeed be observed by a Doppler radar when there are insects and other passive material targets in the lower troposphere [37,89]. However, it is important to note that the duration and the horizontal and vertical scales of gust fronts are much larger than the gusts addressed in this paper. In [37,89], they lasted for several minutes, had a horizontal extent of a few kilometers and encountered a depth from a few hundred meters up to two kilometers or even more. These types of gravity-driven gust fronts associated with downdrafts from deep convection cannot be regarded as typical atmospheric boundary-layer wind gusts, but require different spatial and temporal scales to represent their physical processes and their effect on structures.

## 5. Airborne Measurements

### 5.1. Research Aircraft

**Figure 7:**
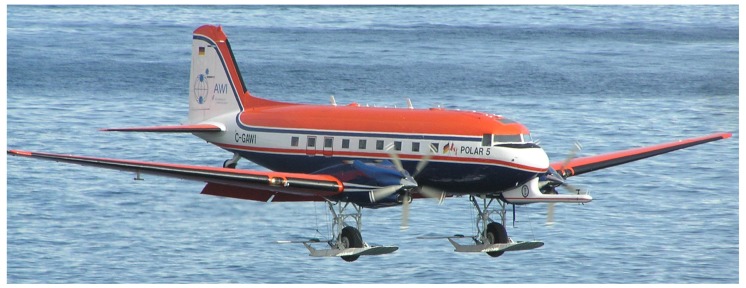
Polar 5 research aircraft of Alfred Wegener Institute, Germany.

Research aircraft, such as the one in Figure 7, have been used for turbulence measurements already for decades, but only recently have wind gusts been assessed based on this type of measurement [38]. Suomi et al. (2016) investigated the possibilities of estimating wind gusts from turbulence measurements taken on board a research aircraft under the assumption that the gusts can be produced by many different factors, for instance turbulent fluctuations or intermittency. Research aircraft measure over a fairly long distance during a short time interval. Therefore, the definition of a gust can no longer be based on temporal, but instead on spatial averaging. To derive the length scales $x_{g}$ and *X* corresponding to the time scales $t_{g}$ and *T* (Section 2), four different methods were tested. One of the methods was based on Taylor’s hypothesis of frozen turbulence [90], which assumes that a stationary wind field is advected past a fixed point by the mean wind speed *U*. Then, the relationship between the distance and the time becomes x=Ut. Another method used by [38] to convert between the scales is based on the assumption that the sampling frequencies represent the same scales of motion. If the sampling frequency of the five-hole probe on board the research aircraft was $f_{o,a}$ and the sampling frequency at a fixed point $f_{o,p}$, then with a ground speed $V_{a}$ of the aircraft, the length scale becomes $x=\frac{f_{o,p}}{f_{o,a}}V_{c}t$. The third method used by [38] to derive the length scales was just to assume that the ratio of $t_{g}$ and *T* is equal to the ratio of $x_{g}$ and *X*.

Suomi et al. (2016) [38] further developed a new method to convert between temporal and spatial scales. It is based on the comparison of median peak factors from a research aircraft and from a meteorological mast. Although the datasets used by [38] were from different locations (research aircraft measurements from the marine Arctic and the meteorological mast measurements from over flat grassland terrain in Denmark) and periods (spring 1998 and full year 2010, respectively), it was possible to apply the method, because it is assumed that statistically, the behavior of the time series in an ensemble of samples does not depend on location or on the period, if the conditions are homogeneous and stationary (Section 2).

**Figure 8:**
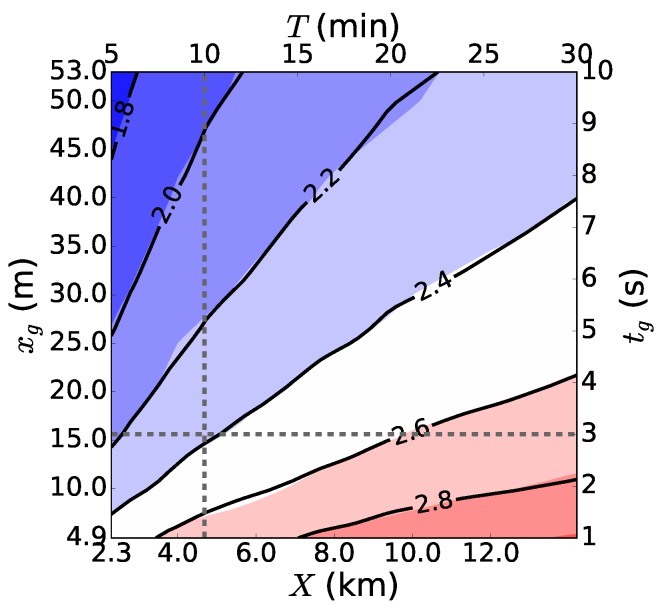
Median peak factor distribution as a function of the time scales $t_{g}$ and *T* (contours) and the length scales $x_{g}$ and *X* (colors). The mast measurements shown as a function of the time scales were taken at the 40-m level of the Høvsøre meteorological mast. The length scale counterparts were derived from all the low level flights during the research aircraft campaign. The length scales ($x_{g}$,*X*) corresponding to the time scales ($t_{g}$,*T*) are (15.6 m, 4.7 km). Figure reproduced with permission from [38].

To compare the statistical behavior of peak factors from both data sources, median peak factors were computed with different combinations of [$x_{g}$,*X*] and [$t_{g}$,*T*] from the research aircraft data and from the meteorological mast measurements, denoted here as $g_{x}\left(x_{g},X\right)$ and $g_{x}\left(t_{g},T\right)$, respectively. A fixed range of temporal averages was set for $t_{g}$ from 1–10 s and for *T* from 5–30 min. Corresponding ranges of the length scales $x_{g}$ and *X* that minimize the difference between $g_{x}\left(x_{g},X\right)$ and $g_{x}\left(t_{g},T\right)$ in the least squared sense were then determined. One of the resulting fitted field pairs is shown in Figure 8. From this, it is possible to find the length scale counterparts for any pair of gust time scales. For example, in Figure 8, the time scales $t_{g}=$ 3 s and T= 10 min correspond to length scales $x_{g}=$ 15.6 m and X= 4.7 km.

**Figure 9:**
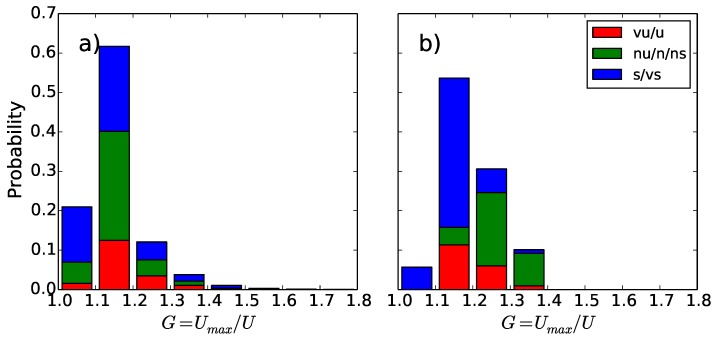
Histograms of *G* observed at the 62-m level in a meteorological mast over the Baltic Sea about 8 km south of Helsinki, Finland (**a**), and *G* based on horizontal low-level flights over Arctic sea ice and open water (**b**). Stability groups are defined as vu/u: very unstable/unstable; nu/n/ns: near neutral unstable/neutral/near neutral stable; vs/s: very stable/stable. Stabilities are defined as in Figure 6. The results are based on the Taylor hypothesis [90]. Redrawn with permission from [40].

Sensitivity tests showed that by choosing data from different heights of the meteorological mast, the gust length $x_{g}$ increases with increasing mast height, whereas the sample length *X* decreases with height. The first result was an expected one, since the integral length scale of the horizontal wind speed typically increases with height [91]. However, in eddy covariance calculations, a longer sample length *X* is usually required at higher measurement heights in order to cover all the scales of turbulent motion contributing to the flux. Because of this discrepancy, Suomi et al. (2016) [38] used the results following from Taylor’s hypothesis to determine the gust length scales. Assuming a constant advection speed of 10 m s${}^{-1}$, they were: $x_{g}=$ 30 m and X= 6 km. Figure 9 illustrates how gust factors calculated using these length scales (based on data from the marine Arctic over open water and sea ice) compare well with independent meteorological mast measurements over open water in the Baltic Sea. In other words, the length scales were chosen successfully. However, it is noteworthy that the difference between these results and those from the peak factor method ($x_{g}=$ 15.6 m and X= 4.7 km) are not large in terms of *G*. Using the median peak factors from both length scale pairs (($x_{g}=$ 30 m, X= 6 km) and ($x_{g}=$ 15.6 m, X= 4.7 km)) and assuming a turbulence intensity I=0.2, which is a very high value [92], the difference in *G* is only about 1.7%, which is small compared to the other factors affecting *G*: the surface roughness, stability and height above the surface. In the future, more research is needed to better understand the results from the novel peak factor method and how it could be applied further.

### 5.2. Unmanned Aircraft Systems

Unmanned aircraft have been used for military purposes already for a century [93], but instrumented UASs have become popular in meteorological research only during the last few decades. There exists a wide range of different types and sizes of UASs, their weights ranging from a few hundred grams to several thousands of kilograms, operating altitudes from a few tens of meters to 20 km and operating distances from a few kilometers to several thousands of kilometers [93]. In meteorological research, the typical UASs comprise relatively small aircraft and rotorcrafts, which are either ground-controlled or semi-autonomous (e.g., [94,95]), but also massive UAS, such as the Global Hawk, has been applied [96].

The wind speed measurements on board an unmanned aircraft can be performed based on instrumentation carried by the aircraft (typically a five-hole probe) or indirectly from the attitude and position records based on the inertial measurement unit (IMU) and GPS, respectively [95]. Yet, wind gusts have not been observed based on unmanned aircraft, but probably, a similar method as for manned research aircraft [38] could be applied. However, due to the generally lower flight velocities (ground speed) of small and medium-size UASs (up to about 30 ms${}^{-1}$) compared to manned research aircraft, the measured wind signals are also affected by temporal changes in the wind velocity. Moreover, the speed and the size of the aircraft may pose limitations to the wind velocity measurement range. Reuder et al. (2012) report that their small unmanned meteorological observer (SUMO) provides wind measurements comparable to radio soundings or tethered balloons, but is not suitable for extreme wind conditions [94]. The success of UAS measurements in strong wind conditions depends on the size of the aircraft, and therefore, most UASs are not best suited for extreme gust conditions when wind gust speeds exceed the UAS operational speeds. The largest UASs, however, have potential to provide wind gust observations in conditions too dangerous for manned aircraft.

Unmanned rotorcrafts, small remotely-piloted copters with multiple rotors, can also be used to measure wind. However, also they are suitable to be used in light and moderate winds, not in extreme gust conditions. The wind can be measured directly by the instrument carried by the copter, like by a sonic anemometer, or indirectly from the attitude of the copter [97].

### 5.3. Other Airborne Techniques

In extremely strong wind conditions, such as tornadoes and tropical cyclones, all measurement techniques presented so far have constraints. Although sonic anemometers can measure winds up to about 33–65 ms${}^{-1}$ [98,99] and cup anemometers up to 75 ms${}^{-1}$ [100], the reported extreme gusts seldom exceed 40–50 ms${}^{-1}$ because of power outages and/or anemometer or supporting structure failures caused by wind-borne debris [101,102]. An alternative method to measure extreme winds is dropwindsonde, which measures the wind based on the location of the sonde tracked by GPS [103]. Wind speed can be measured with an accuracy of 0.5–2 ms${}^{-1}$ and a vertical resolution of about 5 m along the path across the entire troposphere from about a 12-km height down to 4–10 m above the sea surface (for safety reasons, they can only be operated over the sea), and the measured wind speed range is up to 150 ms${}^{-1}$. Dropwindsondes can provide unique information for example of high wind speeds in tropical cyclones [104]. Although these measurements can provide information on the most extreme winds in the atmosphere, the measurements represent the (almost) instantaneous state of the atmosphere similar to radio soundings and therefore do not necessarily capture the absolute maximum wind speed at each measurement height. In other words, the measured wind speeds do not correspond to wind gusts as defined in this work (Section 2).

## 6. Reporting Practices

The World Meteorological Organization (WMO) provides guidelines for meteorological observations and their international exchange, which has been based on alphanumeric codes that have later been replaced mostly by binary formats. WMO recommends measuring wind gust speed as a 3-s average of wind speed during a 10-min sampling interval [29]. However, there exists differences in both measurement and reporting practices, and these may vary at the regional and national level.

The traditional WMO alphanumeric codes for surface observations are called SYNOP (surface synoptic observations; fixed land stations), SYNOP MOBIL (mobile land stations) and SHIP (ship synoptic observations; sea stations) [105]. In general, these codes do not include wind gusts, although deviations from the standards exist at the national level (for example, in Argentina, the maximum hourly gust speed is reported if the observed gust is equal to or higher than 30 knots (≈15 ms${}^{-1}$) [106]). The codes called METAR (METeorological Aerodrome Report) and SPECI (aviation SPECIal weather report) used for aerodrome routine reports include gusts, but they are reported only if the wind gust speed, defined as a 3-s average, exceeds the 10-min mean wind speed by 5 ms${}^{-1}$ (10 knots) or more [105].

The binary formats regulated by WMO are called BUFR (Binary Universal Form for the Representation of meteorological data) and GRIB (GRIdded Binary or General Regularly-distributed Information in Binary form) [107]. The latter comprises regularly-distributed information such as gridded data from NWP systems. BUFR, on the other hand, is a binary universal form for the representation of meteorological data and is used for the exchange of surface observations, but also of many other types of meteorological measurements and forecast information. In BUFR format, wind gusts can be reported both from land and sea stations (SYNOP and SHIP stations), as well as from aerodrome reports (METAR/SPECI).

Despite the WMO recommendations for wind gust measurements, the measurement and recording practices may vary regionally and nationally. For example, in the ASOS network in the USA, wind gusts are recorded as the maximum 5-s average of wind speed during the past 10-min period preceding the time of observation. At the same time, the averaging time for the mean wind speed is 2 min instead of 10 min. Moreover, wind gusts are reported only if the highest gust during the past 10 min exceeds the 2-min mean wind speed by at least three knots, given that the mean wind speed is greater than two knots, and the highest gust exceeds the minimum five-second wind speed during past 10 min by 10 knots or more. In other words, the minimum reported wind gust speed is 14 knots. In addition, the peak wind may be reported as the highest 5-s average of wind speed that exceeds 25 knots since the last hourly (METAR) observation [108]. Although at ASOS stations, the wind gust duration has traditionally been 5 s instead of 3 s, at some locations, the 3-s gust duration was adopted later [42].

Different measurement and recording practices make the intercomparison of observed wind gusts challenging. Therefore, it is crucial that the averaging periods are well documented and available for data users. For example, if the wind gusts are reported only when a certain threshold value is exceeded, the resulting wind gust statistics are not representative of the actual wind gust climate observed at the measurement site. However, if the wind gust speed is recorded only during the last 10-min period preceding the observation time at each hour, the resulting gusts will always be equal to or weaker than the true maximum wind gust during the preceding hour [42]. This is of particular concern in design load calculations, where the aim is to estimate the probability of occurrence of extreme wind gusts with return periods typically from 10–50 years or more.

## 7. The Effect of Environmental Conditions on Wind Gust Measurements

The overall accuracy of wind measurements includes the accuracy of the data acquisition system and the effect of environmental conditions. Especially in climatological studies, the overall accuracy is highly important, because the inter-decadal trends in wind speed are typically of the order of only 0.5 ms${}^{-1}$ [31]. Standard in situ anemometers used at weather stations can provide measurements with high enough precision, but the effect of environmental conditions may become critical for the measurement accuracy. Large obstacles in the vicinity of anemometers can cause a wake that is 2.5-times the height of the obstacle in near-neutral stability conditions, but in stable conditions, the wake can reach much further (e.g., [109]).

As pointed out already in Section 1, exposure corrections can be applied to correct mean wind speed measurements for suboptimal conditions [34,35,36]. However, these methods are not suitable for correcting wind gusts. Another possibility to obtain more representative measurements is to increase the measurement height from the standard 10-m level [29]. The effect of measurement height and surface roughness on the gust factor can be demonstrated by assuming neutral atmospheric conditions with a logarithmic wind profile:

(23) $$U\left(z\right)=\frac{u_{*}}{\kappa }\ln\left(\frac{z}{z_{0}}\right),$$

where $u_{*}$ is the friction velocity, κ the von Karman constant, *z* the height above surface and $z_{0}$ the aerodynamic roughness length. Assuming $\sigma_{u}\approx 2.5u_{*}$ and combining Equation (23) with Equations (1) and (22) yield:

(24) $$G=1+g_{x}\frac{1}{\ln\left(z/z_{0}\right)}.$$

**Figure 10:**
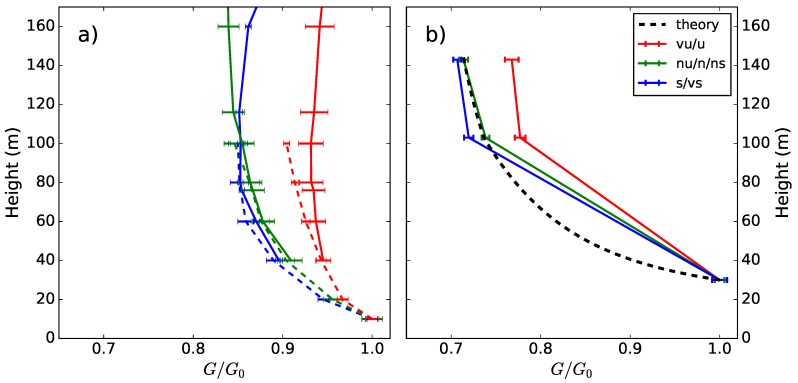
Mean gust factor profiles over (**a**) smooth grassland and (**b**) rough forest surface. In (a), the dashed lines are based on sonic anemometer data from a meteorological mast, and the solid lines are from the corresponding data from a Doppler lidar. The theoretical curve in (b) is based on Equation (24) with the height *z* replaced by z−d, where d= 11 m is the displacement height. The roughness length is $z_{0}=$ 2.5 m. Gust factors are normalized by the value at the lowest level. Error bars show the standard error of the mean. Stability groups are defined as vu/u: unstable (−200 <L< −50), nu/n/ns: near neutral |L|>200 and vs/s: stable (10 <L< 200). Redrawn with permission from [40].

The dependency of the gust factor on height in optimal conditions over flat grassland terrain and over rough forest surface is shown in Figure 10. In the latter case, also the theoretical profile based on Equation (24) is shown. In the optimal conditions (Figure 10a), the gust factor decreases with height only by about 5–15% above the 10-m level, whereas above a rough forest surface, where the average tree height is about 10–15 m and the lowest measurement height is at 30 m, the decrease from the lowest (30 m) to the second-lowest (100 m) measurement level is as much as 20–30%. The theoretical curve shows that the change is the strongest near the surface. In other words, even if the measurements were obtained at a 30-m height instead of 10 m, they are strongly influenced by the roughness elements of the underlying forest, which, for example, affects comparisons with model results, when the model gust factors are based on the average surface conditions in the grid scale, i.e., in the scale of a kilometer or more. As seen in Figure 10a, Doppler lidar can provide information on gust factor profiles and could therefore be a good tool to investigate the optimal measurement height for wind gusts when the environment surrounding the weather station is not optimal.

## 8. Conclusions and Outlook

The aims of this work were as follows: (1) to give an overview of traditional wind gust measurement techniques applied in meteorology and some historical insight to their development; (2) to present recent developments to measure wind gusts based on Doppler lidar and research aircraft data; and (3) to discuss the effects of environmental conditions on measuring wind gusts.

Wind gusts are measured operationally at weather stations all over the world. The measurements are taken applying mainly cup and sonic anemometers, which in ideal conditions are placed at a 10-m reference height. The practices related to the measurements and international data exchange are coordinated by WMO, who recommends measuring the wind gust speed as a 3-s maximum of wind speed during a 10-min sampling period [29]. However, in practice, there are regional differences both in sampling and reporting practices. Gusts may be reported only when a certain threshold value is exceeded or only during the 10-min period prior to the hourly observation time. Then, the resulting wind gust time series have gaps that may cause errors in estimating return periods for extreme wind loads necessary for structural design and, for example, in the insurance field. Moreover, the overall accuracy of the measurements includes not only the accuracy of the anemometer system, but also the environmental conditions. The changes in the environmental conditions surrounding the weather station may cause artificial changes in the measured wind gust climate.

The weather station measurements have many limitations. The environmental conditions may be suboptimal due to shadowing effects of obstacles nearby the anemometer. Sometimes, a better representativeness of the observations is obtained by increasing the measurement height, but even then, the measurements are point-wise, and no information is available on the horizontal and vertical variability of wind gusts. Moreover, the weather stations are typically located in places were they are easy to apply. The majority of stations are located over densely-populated land areas, whereas in remote locations such as the marine Arctic, only a few stations exist, and those are mainly located at sparse coastal locations.

New measurement techniques based on remote sensing and airborne measurements can provide valuable information on wind gusts complementing that based on the existing weather station network. Exploitation of these measurements will be a challenge in the forthcoming years.

Doppler lidars can provide valuable information on wind gust profiles. Such information could be used, for example, to find out the optimal measurement height and location for the traditional anemometer at a weather station. Doppler lidars could also be utilized to investigate the formation and the origin of wind gust events, which could further be used to develop parameterizations for NWP and climate models to forecast wind gusts. Existing parameterizations can be divided into surface-based methods and a profile method. Surface-based methods aim at estimating the gust factor, which is the ratio of the wind gust speed to the mean wind speed. It depends on the height above the surface, surface roughness and the static stability of the atmosphere. Parameterizations applied in NWP models can take these observed factors into account [45,53,110,111]. However, based on in situ measurement techniques, it has not been possible to compare the surface-based wind gust forecasting methods with the so-called profile method by Brasseur (2001) [112], which has been applied in many NWP studies [113,114,115,116,117], but never assessed based on observations alone. The method assumes that wind gusts observed near the surface are caused by large turbulent eddies bringing high-momentum air aloft down to the surface. An observational study to investigate the validity of this theoretical method would require simultaneous profiles of the mean wind speed, TKE and buoyancy (temperature, humidity). In the future, Doppler lidar technology may provide a possibility for direct evaluation of the Brasseur method with observations, provided that a temperature profile is available simultaneously, for example from a tethered balloon or from UAS (e.g., [118]). In the future, the comparison of gusts from a lidar with model results from Large Eddy Simulations (LES) can potentially provide a possibility to better understand the mechanisms by which the gusts are generated in the atmosphere, and thereby, also new parameterizations for NWP models can potentially be developed to improve wind gust forecasting.

The theoretical approach to estimate wind gusts, the so-called Rice theory, has many practical applications. It can be used to convert peak and gust factors when observations are made with different gust time scales $t_{g}$ and *T* [46]. It can also be applied to obtain improved wind gust measurements from a Doppler lidar, which has a limited sampling frequency and the measurements include spatial averaging. In the future, the next step will be to test the applicability of the method to other lidar types and scanning sequences, such as conical scans with many more beams. An open question is, for example, what is a sufficient Doppler lidar measurement frequency for obtaining reliable wind gust estimates? Furthermore, it is important to understand how the horizontal heterogeneity affects the measurements. To understand that, measurements from various environments are needed.

Research aircraft can provide information from remote locations, where deployment of other type of wind instrumentation is challenging or even impossible. However, research aircraft are mostly applied in specific campaigns, and even in the future, we cannot expect a high spatial and temporal coverage of observations. However, with the rapidly developing UAS technology, there are better perspectives for extensive gust observations applying UASs.

A new application of the peak factor theory is the comparison of temporal and spatial scales used to define wind gusts when measurements are available both from a fixed position (meteorological mast) and a moving platform (research aircraft). The method compares directly the statistical properties of the time series without a need for assuming an advection velocity, which is a prerequisite of the well-known Taylor’s hypothesis of frozen turbulence [90]. In fact, the peak factor method can be applied when Taylor’s hypothesis is not valid, for example when the mean (advection) velocity is zero. The method could thus provide new insight to understand turbulence in low-wind conditions typical for a stable atmospheric boundary layer. An open question is, for example, could the peak factor method provide new information on how the turbulent time and length scales compare in non-stationary wind speed conditions in different environments and stability conditions, provided that the time series from a fixed point represents the same environment (and period) as the data in terms of distance. Besides being based on measurements, these types of tests could also be based on LES results or on measurements from a wind tunnel where the degree of nonstationarity in the flow field can be controlled.

In this paper, we have reviewed the past and present wind measurement techniques from the perspective of wind gusts. In order to measure the short-duration wind speed maxima, a high sensitivity of the anemometer combined with a high measurement rate are needed. Moreover, the anemometer must be robust to bear even the most extreme winds. To achieve these goals, the current measurement techniques are based on a long history of development of the methods. Regular observations are made in a worldwide network of weather stations, yielding large amounts of data. In addition, new observation techniques and data analysis methods are actively developed. Due to the combination of large data availability and recent and ongoing methodological developments, we can expect a major advance in the understanding and prediction of wind gusts in the future. This might be useful for many practical applications, and could help in foreseeing the effect of extreme wind loads, which can cause damage and risks to safety.
